# Supplementary material for: Silicon: quantum dot photovoltage triodes
Source: Nat Commun. 2021 Nov 18;12:6696. doi: 10.1038/s41467-021-27050-9 (PMC8602655; doi:10.1038/s41467-021-27050-9)
Supplement: Supplementary file 1 — Supplementary Information [file 41467_2021_27050_MOESM1_ESM.pdf]

Supplementary Information

Silicon: Quantum Dot Photovoltage Triodes

Wen Zhou[1], Li Zheng[1]*, Zhijun Ning[2]*, Xinhong Cheng[1], Fang Wang[3], Kaimin Xu[2], Rui Xu[2], Zhongyu Liu[2], Man Luo[3], Weida Hu[3], Huijun Guo[3], Wenjia Zhou[2], Yuehui Yu[1]

[1]State Key Laboratory of Functional Materials for Informatics, Shanghai Institute of Microsystem and Information Technology, Chinese Academy of Sciences, Shanghai 200050, P. R. China

[2]School of Physical Science and Technology, ShanghaiTech University, Shanghai 201210, P. R. China

[3]State Key Laboratory of Infrared Physics, Shanghai Institute of Technical Physics, Chinese Academy of Sciences, Shanghai 200083, P. R. China

[*]Corresponding author: zhengli@mail.sim.ac.cn, ningzhj@shanghaitech.edu.cn

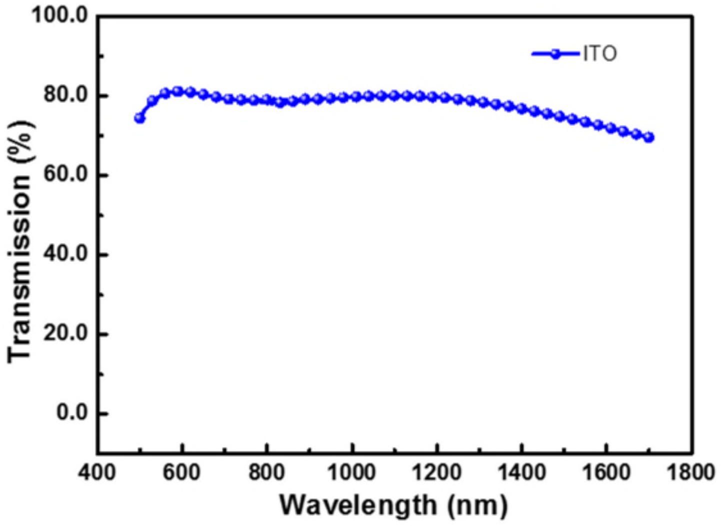

Supplementary Figure 1. Transmission spectrum of ITO. The ITO electrode has high transmission of ~75% at the wavelengths from 500 nm to 1700 nm.

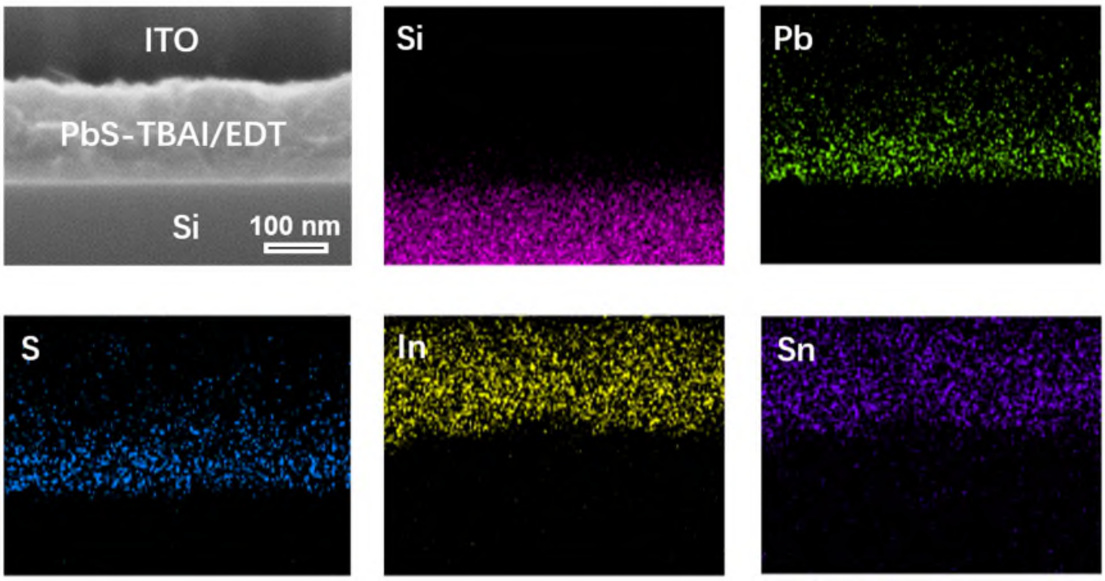

Supplementary Figure 2. Energy dispersive X-ray spectroscopy (EDS) of the Si:PbS PVTRI with an ITO electrode. The main elements of each layer are labeled by different colors.

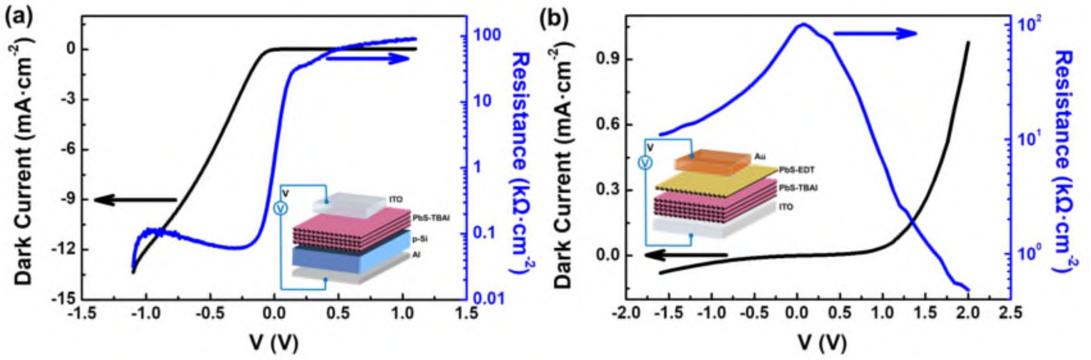

Supplementary Figure 3. The junction characteristics of the p-Si:n-CQD heterojunction and the n-CQD:p-CQD junction. (a) The p-Si:n-CQD heterojunction is consisted of the 0.16 μm PbS n-CQD and the p-Si substrate. ITO and Al serve as the top and bottom electrodes, respectively. The heterojunction shows an excellent rectifier characteristic. (b) The n-CQD:p-CQD junction is consisted of the 0.16 μm n-CQD and 0.04 μm p-CQD. ITO and Au serve as the top and bottom electrodes, respectively. This junction also has a rectifier characteristic.

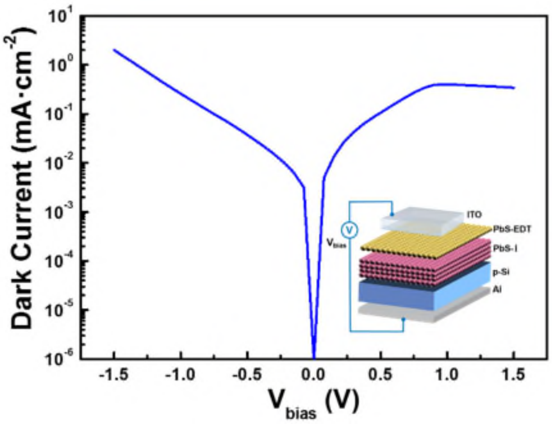

Supplementary Figure 4 The dark current of our PVRTI. The dark current densities are 10^{-5}-10^{-3} A·cm^{-2} (10^{-2}-10^{0} mA·cm^{-2}) for a bias voltage of V_{bias}= -0.5 V to -1.5 V, which are four orders of magnitude lower than that of previous infrared sensitized silicon detectors[1].

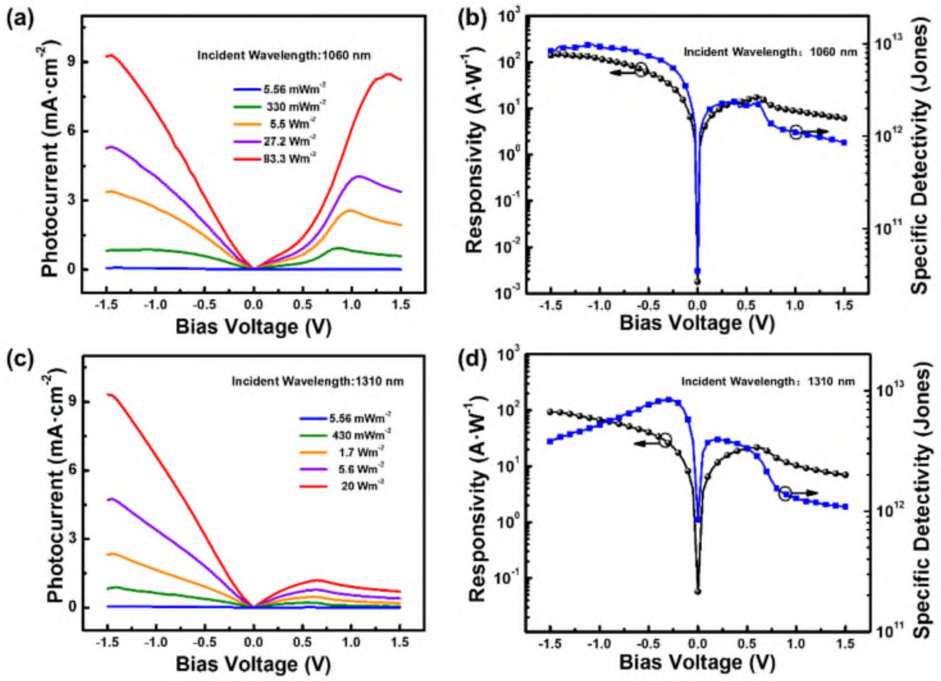

Supplementary Figure 5. The operational behaviors of our PVTRI under illumination of 1060 nm and 1310 nm. (a) Output characteristics of the PVTRI under 1060 nm illumination with different incident powers. The tendency of the photocurrent is similar to the one measured under 637 nm illumination. (b) The detected maximum responsivity is 146 $A \cdot W^{-1}$ and specific detectivity is ~10^{13} Jones. (c) Output characteristics of the PVTRI under 1310 nm illumination with different incident powers. The tendency of the photocurrent is similar to the one measured under 1550 nm illumination. (d) The detected maximum responsivity is in the order of magnitude of 10^2 $A \cdot W^{-1}$ and specific detectivity is close to the order of 10^{13} Jones (8.4×10^{12} Jones).

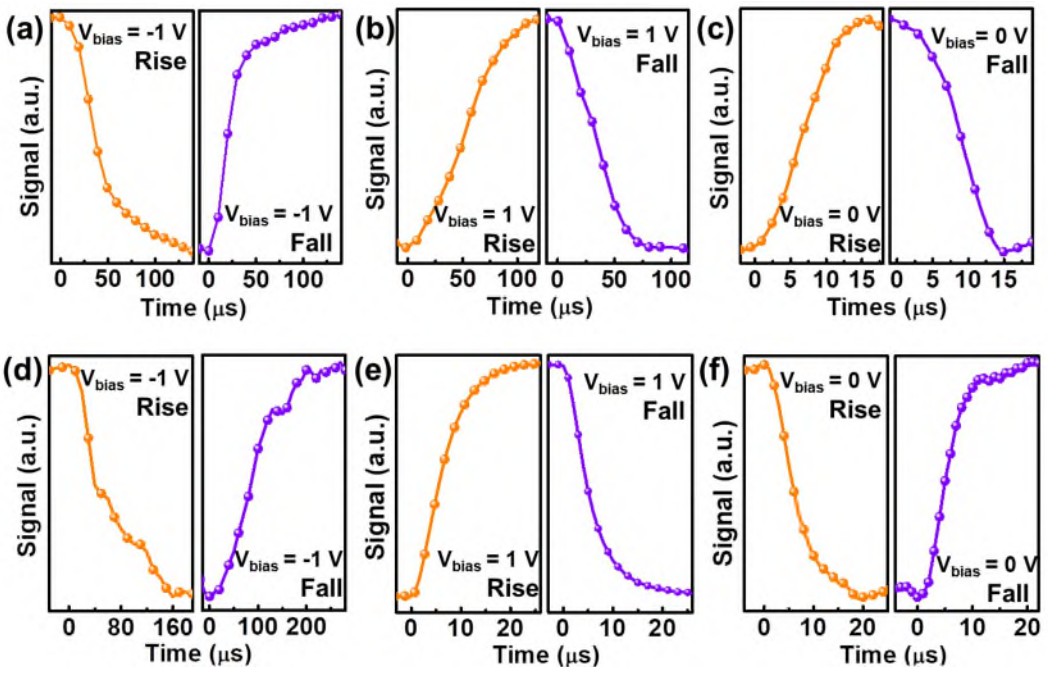

Supplementary Figure 6. Response time of the PVTRI under illumination of (a-c) 1,550 nm and (d-f) 637 nm corresponding to Figure 5. The fast components of the rise and fall time at V_{bias} = -1 V (a, d), 1 V (b, e) and 0 V (c, f). a.u. means arbitrary units.

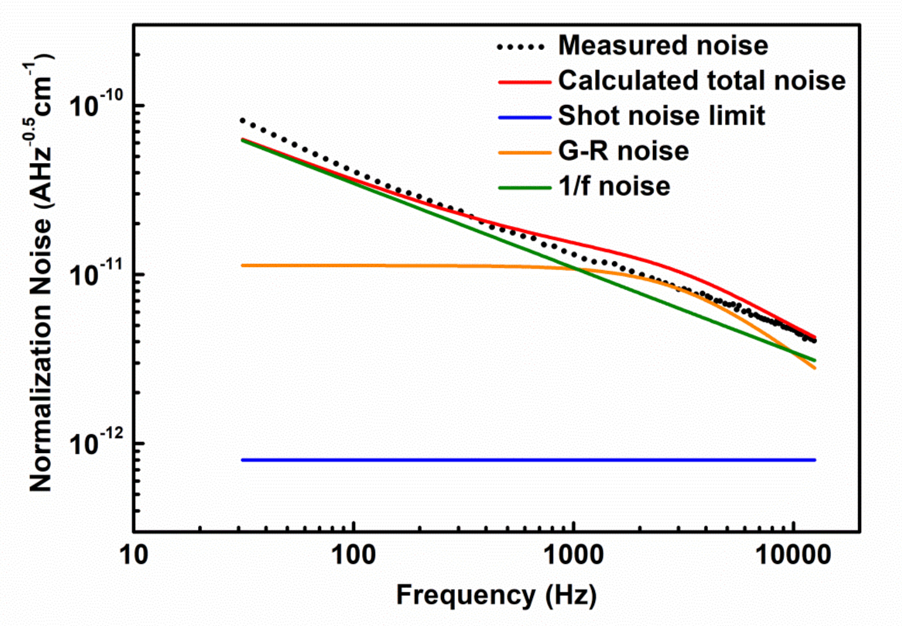

Supplementary Figure 7 Normalization noise current (I_n^*) was measured as a function of frequency at bias voltage V_{bias} = -0.4 V for the PVTRI. The noise spectrum of a detector is a plot of noise current spectral density versus frequency that is used to determine the magnitude of noise at the frequency at which the photodetector operates[2]. The measured noise, calculated 1/f noise, shot noise, and G-R noise limit are also included for reference.

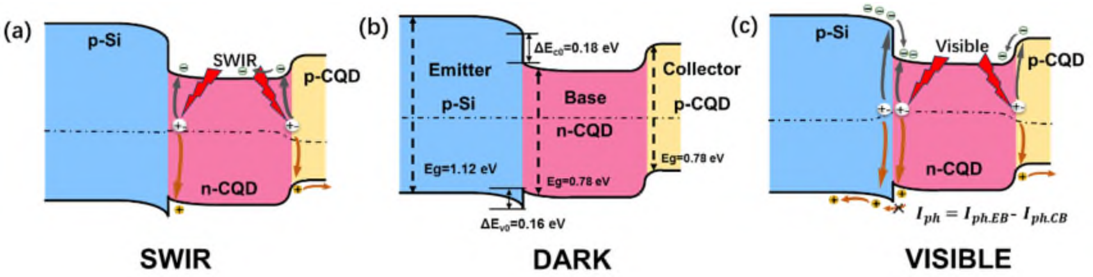

Supplementary Figure 8. Energy bands and operation mechanism of the PVTRI with the zero bias voltage. (a) Excitation of SWIR, (b) in the dark and (c) excitation of visible lights.

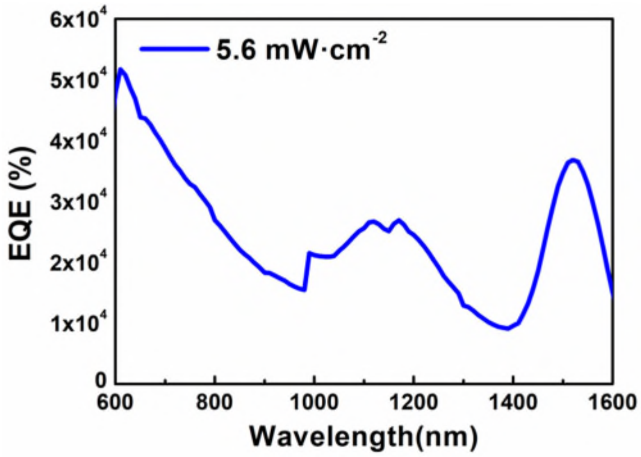

Supplementary Figure 9. Spectral EQE plot of the PVTRI.

Supplementary Note 1. Calculation of the energy band barriers of the Si:n-PbS heterojunction.

The conduction band energy level (E_{CB}) of n-PbS can be calculated and measured by the cyclic voltammetry (CV) method according to the following equation:

$$E_{CB} = 4.5 + 0.799 + 0.059 \log(c_{Ag+}) + E_{RE} \quad (1)$$

where E_{RE} is the reduction potential which can obtain by the CV method as reference[3-4]. c_{Ag+} is the $Ag+$ concentration of the $Ag|Ag+$ electrode that is 0.01 in this work. The E_{RE} value of the n-PbS CQD is measured to be -0.955 eV relative to Ag|Ag+ shown in **Fig. S9a**. The E_{CB} value is calculated to be -4.23 eV.

The bandgap (E_g) of PbS CQDs can be calculated to be 0.78 eV by Tauc plots[5-6] of the PbS CQDs absorption spectrum shown in **Fig. S9b**. The valence band energy (E_{VB}) of n-PbS can be obtained by Eq. 2:

$$E_{VB}=E_{CB}-E_g \quad (2)$$

where the E_{VB} value of the n-PbS CQD is -5.01 eV.

The E_{CB} and E_{VB} of silicon are -4.05 eV and -5.17 eV, respectively[7]. The valence (ΔE_{vo}) and conduction (ΔE_{co}) band offsets between n-PbS and p-silicon are calculated by the Eqs. 3 and 4, respectively.

$$\Delta E_{co}=\Delta E_{CB\_Si}-\Delta E_{CB\_PbS} \quad (3)$$

$$\Delta E_{vo}=\Delta E_{VB\_Si}-\Delta E_{VB\_PbS} \quad (4)$$

which are calculated to be 0.18 eV and 0.16 eV, respectively.

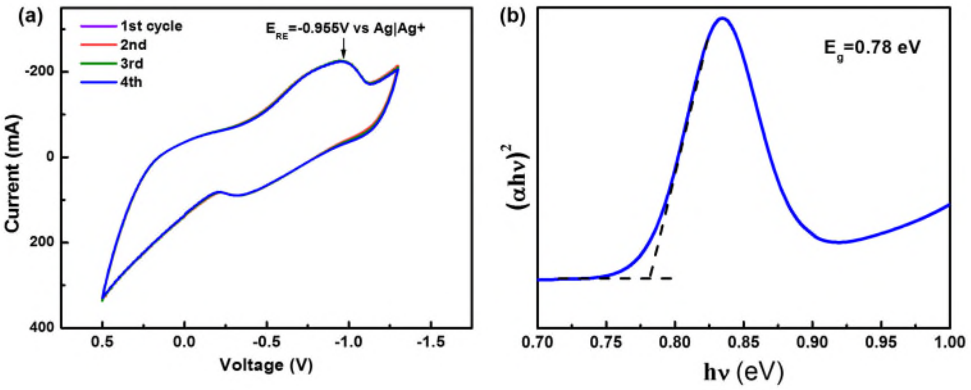

Supplementary Figure 10. Energy bands measurement of n-PbS CQDs. (a) Cyclic voltammetry characterization; (b) Tauc plots calculated form the absorption spectrum.

Supplementary Note 2. Theoretical calculation of the dark current and gain

As for a triode, the small-signal common-base gain β is difined as

$$\beta = \frac{J_{\mathrm{pE}} - J_{\mathrm{rB}}}{J_{\mathrm{nE}} + J_{\mathrm{rB}}} \tag{5}$$

Ignoring the base region recombination current, eq. 5 can be derived as

$$\beta \approx \frac{J_{\mathrm{pE}}}{J_{\mathrm{nE}}} \tag{6}$$

where J_{pE} is the current density due to the diffusion of the minority carriers (holes) in the base, J_{nE} is the current density due to the diffusion of the minority carriers (electrons) in the emitter.

As for a p-n-p triode, J_{pE}, J_{nE} and the reverse-biased saturation current density in the base-collector junction (J_{S}) can be expressed by the following equations[8]

$$J_{\mathrm{pE}} = \frac{\mathrm{e}D_{\mathrm{B}}n_{\mathrm{B0}}}{L_{\mathrm{B}}}\left(\frac{1}{\sinh\frac{x_{\mathrm{B}}}{L_B}} + \frac{\mathrm{e}^{\frac{\mathrm{e}V_{\mathrm{BE}}}{k_{\mathrm{B}}T}} - 1}{\tanh\frac{x_{\mathrm{B}}}{L_{\mathrm{B}}}}\right) \approx \frac{\mathrm{e}D_{\mathrm{B}}n_{\mathrm{Bi}}^2}{L_{\mathrm{eff-B}}N_{\mathrm{B}}}\mathrm{e}^{\frac{\mathrm{e}V_{\mathrm{BE}}}{k_{\mathrm{B}}T}} - 1 \tag{7}$$

$$J_{\mathrm{nE}} = \frac{\mathrm{e}D_{\mathrm{E}}n_{\mathrm{E0}}}{L_{\mathrm{E}}}\frac{\mathrm{e}^{\frac{\mathrm{e}V_{\mathrm{BE}}}{k_{\mathrm{B}}T}} - 1}{\tanh\frac{x_{\mathrm{E}}}{L_{\mathrm{E}}}} \approx \frac{\mathrm{e}D_{\mathrm{E}}n_{\mathrm{Ei}}^2}{L_{\mathrm{eff-E}}N_{\mathrm{E}}}\mathrm{e}^{\frac{\mathrm{e}V_{\mathrm{BE}}}{k_{\mathrm{B}}T}} - 1 \tag{8}$$

$$J_{\mathrm{S}} = \frac{\mathrm{e}D_{\mathrm{B}}p_{\mathrm{B0}}}{L_{\mathrm{B}}} + \frac{\mathrm{e}D_{\mathrm{C}}n_{\mathrm{C0}}}{L_{\mathrm{C}}} = \frac{\mathrm{e}D_{\mathrm{B}}n_{\mathrm{Bi}}^2}{L_{\mathrm{B}}N_{\mathrm{B}}} + \frac{\mathrm{e}D_{\mathrm{C}}n_{\mathrm{Ci}}^2}{L_{\mathrm{C}}N_{\mathrm{C}}} \tag{9}$$

where $D_{\mathrm{E/B/C}}$ is the minority carrier diffusion coefficients in the emitter/base/collector, $n_{\mathrm{Ei}}/p_{\mathrm{Bi}}/n_{\mathrm{Ci}}$ is the intrinsic carrier concentrations in the emitter/base/collector, $N_{\mathrm{E/B/C}}$ is the doping concentrations in the emitter/base/collector, and $L_{\mathrm{eff-E/B/C}}$ is effective minority carrier diffusion lengths in the emitter/base/collector.

According to the above equations, β can be written as

$$\begin{aligned}\boldsymbol{\beta} &= \frac{n_{Bi}^2 N_E D_B L_{eff-E}}{n_{Ei}^2 N_B D_E L_{eff-B}} = \frac{(m_{nB}^* m_{pB}^*)^{\frac{3}{2}} N_E D_B L_{eff-E}}{(m_{nE}^* m_{pE}^*)^{\frac{3}{2}} N_B D_E L_{eff-B}} e^{\frac{\Delta E_g}{k_B T}} \\ &\approx \frac{\boldsymbol{N_E D_B L_{eff-E}}}{\boldsymbol{N_B D_E L_{eff-B}}} \boldsymbol{e^{\frac{\Delta E_g}{k_B T}}}\end{aligned} \tag{10}$$

Similarly, the dark current can be written as

$$\boldsymbol{J_{dark}} = (1+\beta)J_{CBO} \approx \boldsymbol{\beta J_S} \tag{11}$$

and the photo gain can be estimated as

$$\boldsymbol{G} = \boldsymbol{\eta} \cdot \boldsymbol{\beta} \tag{12}$$

where η is the external quantum efficiency of the base-collector junction.

i. As for $V_{bias} < 0$ V, e. g. $V_{bias} = -1.5$ V, $V_{\text{bi-CB}}$, $x_{\text{n-CB}}$, $L_{\text{eff-B}}$, $L_{\text{eff-E}}$ and ΔE_{g} can be calculated according to the parameters in Supplementary Table 1

$$V_{bi-CB} = \frac{k_{\text{B}}T}{\text{e}} \ln\left(\frac{N_{\text{C}}N_{\text{B}}}{n_{\text{i}}^2}\right) = 0.24 \text{ V}$$

$$x_{\text{n-CB}} = \left[\frac{2\epsilon_s(V_{\text{bi-CB}} + V_{\text{CB}})}{\text{e}} \frac{N_{\text{C}}}{N_{\text{B}}} \frac{1}{N_{\text{C}} + N_{\text{B}}}\right]^{\frac{1}{2}} = 138 \text{ nm}$$

$$L_{\text{eff-B}} = x_{\text{B}} = x_{\text{B0}} - x_{\text{n-CB}} = 22 \text{ nm}$$

$$L_{\text{eff-E}} = L_{\text{E}} = 592000 \text{ nm}$$

$$\Delta E_{\text{g}} = 0.34 \text{ eV}$$

Considering an external quantum efficiency (η) of 10%-30% in the base-collector heterojunction[9, 10], and then β, J_{dark} and G can be obtained as

$$\beta = \frac{N_{\text{E}}D_{\text{B}}L_{\text{eff-E}}}{N_{\text{B}}D_{\text{E}}L_{\text{eff-B}}} \text{e}^{\frac{\Delta E_{\text{g}}}{k_{\text{B}}T}} = 2550$$

$$J_{\text{dark}} = 0.19 \text{ mA} \cdot \text{cm}^{-2}$$

$$G \approx 300 \sim 800$$

The theoretical calculated value of the gain is completely compatible with the measurements reported in the manuscript.

ii. The dark current and gain at $V_{bias} > 0$ can still be calculated according to eqs. 5-12. It is worthy to mention that as for bulk materials, the $\frac{D_{\text{B}}L_{\text{eff-E}}}{D_{\text{E}}L_{\text{eff-B}}}$ term in eq. 10 is negligible and eq. 10 is simplified to the following expression:

$$\beta = \frac{N_E}{N_B} e^{\frac{\Delta E_g}{k_B T}} \tag{13}$$

If using eq. 13 to calculate the gain, the obtained value will not exceed 1. However, as for the zero dimensional materials (e. g. QDs), the diffusion coefficient and the diffusion length are significantly different from the bulk materials, and they cannot be reduced for the calculation of $\boldsymbol{\beta}$**.** According to the parameters in Table R-1, as for $V_{bias} > 0$ V, e. g. $V_{bias} = 1.5$ V, $V_{\text{bi-CB}}$, $x_{\text{n-CB}}$, $L_{\text{eff-B}}$, $L_{\text{eff-E}}$ and ΔE_{g} can be calculated as follows

$$V_{\text{bi-CB}} = 0.11 \text{ V}$$

$$x_{\text{n-CB}} = \left[\frac{2\epsilon_{\text{C}}\epsilon_{\text{B}}(V_{\text{bi-CB}} + V_{\text{CB}})}{\text{e}} \frac{N_{\text{C}}}{N_{\text{B}}} \frac{1}{\epsilon_{\text{C}}N_{\text{C}} + \epsilon_{\text{B}}N_{\text{B}}}\right]^{\frac{1}{2}} = 57 \text{ nm}$$

$$L_{\mathrm{eff-B}} = x_{\mathrm{B}} = x_{\mathrm{B0}} - x_{\mathrm{n-CB}} = 103 \ \ \mathrm{nm}$$
$$L_{\mathrm{eff-E}} = L_{\mathrm{E}} = 4 \ \ \mathrm{nm}$$
$$\Delta E_{\mathrm{g}} = 0 \ \ \mathrm{eV}$$

Considering an external quantum efficiency (η) of 30%-80% in the base-collector homojunction[11], and then β, J_{dark} and G can be obtained as

$$\beta = \frac{N_{\mathrm{E}} D_{\mathrm{B}} L_{\mathrm{eff-E}}}{N_{\mathrm{B}} D_{\mathrm{E}} L_{\mathrm{eff-B}}} = 283$$
$$J_{\mathrm{dark}} = 0.02 \ \ \mathrm{mA \cdot cm^{-2}}$$
$$G \approx 100 \sim 200$$

The theoretical calculated value of the gain is completely compatible with the measurements reported in the manuscript.

Supplementary Note 3. Noise current evaluation and specific detectivity calculation

We assume the read circuit performance. It is attributed to the high compatibility with Si technology of quantum dots. The detailed explanation of the assumption is as follows.

The specific detectivity of the device is

$$D^* = \frac{\sqrt{A\Delta f}}{NEP} = \frac{R\sqrt{A\Delta f}}{I_{ntotal}} = \frac{R\sqrt{A\Delta f}}{\sqrt{2q(GNDCD \cdot A + I_\emptyset)M^2F \cdot \Delta f + I_{nROIC}^2}} \qquad (14)$$

where I_{ntotal} is the noise current, $GNDCD = \frac{I_{dark}}{M \cdot A}$, M is the gain, F is the excess noise, A is photosensitive element area, $I_\emptyset$ is the background luminous flux, Δf is the noise bandwidth, I_{nROIC} is the circuit noise current. Generally, the background luminous flux is relatively small, about 10^8 photons/S/cm^2. For our device area in the manuscript, the current is in the order of sub femto ampere, which can be ignored. Considering the high compatibility with Si technology of quantum dots and the strikingly similar noise figure of our device and the silicon-only device[1], we assumed that the number of circuit noise electrons stayed below single figures, which can be ignored in the multiplier detector. In these cases, the specific detectivity of the device is

$$D^* = \frac{\sqrt{A\Delta f}}{NEP} = \frac{R\sqrt{A\Delta f}}{I_{ntotal}} = \frac{R\sqrt{A\Delta f}}{\sqrt{2qGNDCD \cdot A \cdot M^2 \cdot F \cdot \Delta f}}$$

$$= \frac{R\sqrt{A}}{\sqrt{2qI_{dark} \cdot M \cdot F}} = \frac{R}{\sqrt{I_n^{*2}}} \qquad (15)$$

Under this condition, the device bandwidth can be ignored.

The maximum specific detectivity extracted by the dark current method is 4.73×10^{13} Jones with V_{bias} of -0.4 V at 1,550 nm, and the corresponding responsivity is 125 A·W^{-1}. The normalization noise current (I_n^*) was measured as a function of frequency at the same bias voltage (V_{bias} = -0.4 V) for the device and extracted at the cut-off frequency (f_T). The cut-off frequency (f_T) of the PVTRI can be calculated by the equation: $f_T = 1/2\pi\tau$, where τ is the response time. For V_{bias} < 0 V, the response time is about 50 μs (Fig. S6a) and the corresponding normalization noise current is 1.17×10^{-11} AHz$^{-0.5}$cm^{-1}. In order to justify this calculation approach, we have separately extracted the normalization shot noise (I_{ns}^*), the generation-recombination (G-R) noise (I_{ngr}^*) and the 1/f noise (flicker noise, I_{nf}^*) according to eqs. 16-18, calculated the total noise current (I_{ntotal}^*) and compared it with the measured one.

$$I_{ns}^* = \sqrt{2qI_d} \qquad (16)$$

$$I_{ngr}^{*} = \sqrt{\frac{4qGI_d}{1 + (2\pi f\tau)^2}} \quad (17)$$

$$I_{nf}^{*} = \sqrt{\frac{cI_d^{\alpha}}{f^{\beta}}} \quad (18)$$

$$I_{ntotal}^{*} = \sqrt{(I_{ns}^{*})^2 + (I_{ngr}^{*})^2 + (I_{nf}^{*})^2} \quad (19)$$

where I_{d} is the dark current at V_{bias} = -0.4 V, G is the photocurrent gain at V_{bias} = -0.4 V under 1550 nm illumination, f is the work frequency of the device, τ is the average carrier lifetime, C, α and β are constant. α and β are typically set at 2 and 1, respectively[8]. As shown in Fig. S7, the calculated total normalization noise current (red curve) is basic anastomotic with the measured one (black dots). In addition, the calculated total normalization noise current at f_T is 1.01×10^{-11} $\mathrm{AHz^{-0.5}cm^{-1}}$, which is very close to the measured one (1.17×10^{-11} $\mathrm{AHz^{-0.5}cm^{-1}}$). As a result, the calculation approach in this work is accurate for D^* estimation and the extracted D^* of the PVTRI is up to 1.07×10^{13} Jones, which is of the same magnitude as the value calculated by the dark current method.

Supplementary Table 1. The parameters of the PVTRI used in the TCAD Sentaurus simulation and calculation[9-16]

	p-Si	PbS-EDT	PbS-TBAI
Bandgap (eV)	1.12	0.78	0.78
Electron Affinity (eV)	4.07	4.23	4.28
Relative Dielectric Constant	11.7	20	20
Minority Carrier Mobility ($cm^2 \cdot V^{-1}s^{-1}$)	1400	7e-7	5.1e-3
Doping Concentration (cm^{-3})	5e15	1e17	-1e17
Minority Carrier Diffusion Coefficients (cm^2/s)	35	1.81e-8	1.32e-4
Effective Minority Carrier Diffusion Length (nm)	592000	4	290
Intrinsic Carrier Concentration (cm^{-3})	1.02 e10	1e14	

Reference:

1. Adinolfi, V., & Sargent, E. H. Photovoltage field-effect transistors. *Nature* **542**, 324-327 (2017).
2. Saran, R., & Curry, R. J. Lead sulphide nanocrystal photodetector technologies. *Nat. Photonics* **10**, 81-92, (2016).
3. Masala, S. et al. The silicon:colloidal quantum dot heterojunction. *Adv. Mater.* **27**, 7445-7450 (2015).
4. Inamdar, S. N. et al. Determination of Band Structure Parameters and the Quasi-Particle Gap of CdSe Quantum Dots by Cyclic Voltammetry. *Chemphyschem* **9**, 2574-2579 (2008).
5. Murphy, A. B. Band-gap determination from diffuse reflectance measurements of semiconductor films, and application to photoelectrochemical water-splitting. *Sol. Energ. Mat. Sol. C.* **91**, 1326-1337 (2007).
6. Xu, K. et al. Inverted Si:PbS colloidal quantum dot heterojunction-based infrared photodetector. *ACS Appl. Mater. Inter.* **12**, 15414-15421 (2020).
7. Cowley, A. M., &Sze, S. Surface states and barrier height of metal-semiconductor systems, *J. Appl. Phys.*, **36**, 3212-3220 (1965).
8. C. Chen, et al. One-dimensional Sb_2Se_3 enabling a highly flexible photodiode for light-source-free heart rate detection, *ACS Photonics*, **7**, 352-360 (2020).
9. Neamen, D. A. Semiconductor physics and devices. *McGraw-Hill*, (2012).
10. K. M. Xu, et al. Inverted Si:PbS colloidal quantum dot heterojunction-based infrared photodetector. *ACS Appl. Mater. Inter.*, **12**, 15414-15421 (2020).
11. X. Xiao, et al. High quality silicon: colloidal quantum dot heterojunction based infrared photodetector. *Appl. Phys. Lett.*, **116**, 101102 (2020).
12. K. Lu, et al. Efficient PbS quantum dot solar cells employing a conventional structure, *J. Mater. Chem. A*, **5**, 23960-23966 (2017).
13. L. L. Hu, et al. Temperature- and ligand-dependent carrier transport dynamics in photovoltaic PbS colloidal quantum dot thin films using diffusion-wave methods. *Sol. Energ. Mat. Sol. C.*, **164**, 135-145 (2017).
14. M. J. Speirs, et al. Temperature dependent behaviour of lead sulfide quantum dot solar cells and films, *Energ. Environ. Sci.*, **9**, 2916-2924 (2016).
15. V. Adinolfi, & E. H. Sargent, Photovoltage field-effect transistors, *Nature*, **542**, 324-327 (2017).
16. O. Voznyy, et al. A charge-orbital balance picture of doping in colloidal quantum dot solids. *ACS Nano*, **6**, 8448-8455 (2012).
